# A critical overview of emotion processing assessment in non-affective and affective psychoses

**DOI:** 10.1017/S204579602400009X

**Published:** 2024-02-15

**Authors:** Irene Gorrino, Maria Gloria Rossetti, Francesca Girelli, Marcella Bellani, Cinzia Perlini, Giulia Mattavelli

**Affiliations:** 1 IUSS Cognitive Neuroscience (ICoN) Center, Scuola Universitaria Superiore IUSS, Pavia, Italy; 2 Section of Psychiatry, Department of Neurosciences, Biomedicine and Movement Sciences, University of Verona, Verona, Italy; 3 UOC Psychiatry, Azienda Ospedaliera Universitaria Integrata (AOUI), Verona, Italy; 4 Section of Clinical Psychology, Department of Neurosciences, Biomedicine and Movement Sciences, University of Verona, Verona, Italy; 5 Cognitive Neuroscience Laboratory of Pavia Institute, Istituti Clinici Scientifici Maugeri IRCCS, Pavia, Italy

**Keywords:** cognitive neuroscience, psychological assessment, psychosis, schizophrenia

## Abstract

**Aims:**

Patients with affective and non-affective psychoses show impairments in both the identification and discrimination of facial affect, which can significantly reduce their quality of life. The aim of this commentary is to present the strengths and weaknesses of the available instruments for a more careful evaluation of different stages of emotion processing in clinical and experimental studies on patients with non-affective and affective psychoses.

**Methods:**

We reviewed the existing literature to identify different tests used to assess the ability to recognise (e.g. Ekman 60-Faces Test, Facial Emotion Identification Test and Penn Emotion Recognition Test) and to discriminate emotions (e.g. Face Emotion Discrimination Test and Emotion Differentiation Task).

**Results:**

The current literature revealed that few studies combine instruments to differentiate between different levels of emotion processing disorders. The lack of comprehensive instruments that integrate emotion recognition and discrimination assessments prevents a full understanding of patients’ conditions.

**Conclusions:**

This commentary underlines the need for a detailed evaluation of emotion processing ability in patients with non-affective and affective psychoses, to characterise the disorder at early phases from the onset of the disease and to design rehabilitation treatments.

Social cognition refers to the mental operations underpinning social interactions, including the perception, encoding, storage, retrieval and regulation of information about oneself and others (Barkl *et al.*, 2014; Brothers, 1990; Gao *et al.*, 2021; Green *et al.*, 2015, 2008). It is a multifaceted construct entailing five main subdomains: theory of mind (the ability to infer other people’s intentions, inclinations and beliefs), social perception (the identification of social context, roles and rules), attributional bias (the tendency to attribute causes of events to external situations or other’s actions), social knowledge (the awareness of roles, rules and goals that characterise social situations and guide social interactions) and emotion processing (the ability to correctly perceive and use emotions) (Green *et al.*, 2008). These processes require the ability to infer the emotions and thoughts of others (Green *et al.*, 2015). To do so, critical information is provided by facial expressions (Barkl *et al.*, 2014; Fusar-Poli *et al.*, 2009), which convey emotional states and influence the generation and regulation of emotions and behaviour in response to these signals; thus, the accurate reading of expressions is crucial for affective communication and emotional bonding (Ekman, 1993).

A link has been established between deficits in facial emotion recognition, social and emotional functioning (Yoo and Noyes, 2016), which not only contribute to the presence of mood alterations (Oldehinkel *et al.*, 2015; Vrijen *et al.*, 2016) but also have negative implications for subsequent treatments (Shiroma *et al.*, 2014). Indeed, several mental disorders, including schizophrenia (Addington *et al.*, 2006; Green *et al.*, 2015) and psychotic disorders (Benito *et al.*, 2013; Ulusoy *et al.*, 2020), are characterised by deficits in facial emotion recognition.

There is robust evidence that people with affective and non-affective psychoses show impairments in emotion perception (Edwards *et al.*, 2002; Kohler *et al.*, 2010; Priyesh *et al.*, 2022; Rocca *et al.*, 2009), experience reduced interpersonal skills (Pinkham *et al.*, 2007) and report social and work difficulties (Addington *et al.*, 2006; Kee *et al.*, 2003). A recent meta-analysis revealed a specific distinction between the two patient groups. Indeed, individuals with affective psychosis demonstrated a greater ability to identify emotional facial expressions compared to non-affective psychosis patients, in particular for emotions of anger, fear and sadness (De Prisco *et al.*, 2023). Importantly, the severity of psychotic symptoms correlates with deficits in emotion processing (Kohler *et al.*, 2000; Schneider *et al.*, 1995), which can be even present in healthy individuals at higher risk of developing schizophrenia based on risk factors such as schizotypal personality traits or genetic susceptibility (Kee *et al.*, 2004; Van’t Wout *et al.*, 2004). Moreover, first episode psychosis patients, in particular non-affective patients, already showed lower ability to label positive and negative emotional prosody, suggesting early disruption in their emotion recognition system (Caletti *et al.*, 2018). Emotional dysfunction may, therefore, represent an index of early signs of the disease (Seiferth *et al.*, 2008), while strengthening the emotion recognition ability may improve prevention and intervention strategies (Comparelli *et al.*, 2013).

As facial emotion recognition skills can be enhanced by training (Combs *et al.*, 2007; Wölwer *et al.*, 2005), much work has been done to characterise the deficit, its relationship to symptoms and neural basis (Marwick and Hall, 2008). Structural and functional anomalies have been found in the insula and amygdala (Crespo-Facorro *et al.*, 2000; Honea *et al.*, 2005; Wright *et al.*, 2000), with evidence of hypoactivation in patients with schizophrenia for fearful compared to neutral faces (Aleman and Kahn, 2005; Delvecchio *et al.*, 2012). This finding suggests that an undifferentiated amygdala response to fearful and neutral faces may hinder their discrimination and lead to misattribution of fear depending on the context (Marwick and Hall, 2008). In contrast, patients with affective psychosis show increased activation of the amygdala and hippocampus, consistent with the notion of greater arousal responses to emotional stimuli (Critchley *et al.*, 2005; Delvecchio *et al.*, 2012; Santos *et al.*, 2010), but also better contextual appraisal compared to schizophrenia patients (Delvecchio *et al.*, 2012; Gerdes *et al.*, 2010). In addition, increased pulvinar activation in affective psychosis has been found, suggesting a greater focus on emotionally salient stimuli from early stages of visual processing (Pessoa and Adolphs, 2010), whereas the emotional dysregulation may be related to deficit of grey matter volume in subgenual anterior cingulate cortex (Maggioni *et al.*, 2017). Taken together, these findings highlight the complex interplay between neural processes, emotional responses and contextual appraisal in individuals with different forms of psychosis. The role of the amygdala in discriminating and attributing emotions, as well as the increased activation observed in affective psychosis, highlights its potential role in the enhance patients’ experience of fear and arousal (Marwick and Hall, 2008; Wright *et al.*, 2000). On the other hand, findings of reduce volume in limbic regions such as the insula and cingulate cortex may underlie difficulties in adaptive responses to emotional stimuli even at the onset of symptoms (Crespo-Facorro *et al.*, 2000; Maggioni *et al.*, 2017). A better understanding of regional variations and their relation to patients’ symptoms is relevant to shed light on the mechanisms underlying emotional deficits and to offer potential paths for tailored interventions in individuals with psychosis.

Notably, emotion processing involves several stages, which are measured by different tasks. Four main levels can be identified: i) unconscious processing refers to stimuli which are potentially accessible to consciousness but are processed in absence of awareness because they are below the threshold of perception (Dehaene *et al.*, 2006; Mattavelli *et al.*, 2019), ii) perceptual sensitivity is the individual threshold at which stimuli can be differentiated from noise or other stimuli (Pessoa *et al.*, 2005), iii) the discrimination requires to distinguish between expressions and iv) recognition further requires to identify the target emotion (Adolphs *et al.*, 2000). The first two levels are tested with forced-choice tasks and using very brief presentation of stimuli (e.g. 10–30 ms target). Discrimination and recognition are instead assessed by identification tasks. While sensitivity depends on sensory and visuospatial processes, emotion recognition also requires the ability to label the correct emotion among several alternatives (Haxby *et al.*, 2002). As the neural pathways underlying perceptual processing and emotion recognition are partially distinct and can be selectively impaired in different neuropsychiatric conditions (Mattavelli *et al.*, 2021; Tamietto and De Gelder, 2010), it is necessary to assess whether impairments occur at early perceptual processing or/and recognition of emotions.

This commentary aims at presenting an overview of the strengths and weaknesses of the available instruments assessing the different stages of facial emotion processing in patients with non-affective and affective psychoses. Since the deficits in social cognition domain are related to psychotic symptoms and quality of life, a deeper characterisation of emotion processing capacity is crucial for a better understanding of the pathologies and to promote personalised interventions.

Up to date, most studies have used emotion recognition tests in schizophrenia (Addington *et al.*, 2008; Pinkham *et al.*, 2007) and bipolar disorder (Benito *et al.*, 2013; Ulusoy *et al.*, 2020). The most reported are the Ekman 60-Faces Test (EK-60F; Young *et al.*, 2002), the Facial Emotion Identification Test (FEIT; Kerr and Neale, 1993) and the Penn Emotion Recognition Test (ER-40; Kohler *et al.*, 2003). In the former, participants are shown a series of 60 black-and-white photographs of male and female faces expressing one of six basic emotions (surprise, happiness, fear, disgust, anger and sadness) and are required to associate the correct label to each emotion. The reliability and validity of this instrument have been demonstrated in several studies (Róza *et al.*, 2012). The FEIT similarly uses the stimuli developed by Ekman and Friesen (1976) and Izard (1971) but presents only 19 faces, 15 expressing negative (anger, sadness, fear and shame) and 4 expressing positive emotions (happiness and surprise). The FEIT has established psychometric properties (Dougherty *et al.*, 1974; Feinberg *et al.*, 1986; Zuroff and Colussy, 1986) and includes a control task to rule out the possibility that the poor performance of patients generally reflects cognitive impairments (Chapman and Chapman, 1973, 1978; Oltmanns and Neale, 1978). It has been validated for Korean (Bahk *et al.*, 2015) and Chinese (Lo and Siu, 2018) populations, but no normative data are currently available for the European and South American populations, which limits its clinical use. In contrast, the EK-60F has been validated in Korea (Kim *et al.*, 2017) and Italy (Dodich *et al.*, 2014). Finally, the ER-40 includes 40 colour-posed facial expressions of four emotions (anger, sadness, happiness or fear) with high and low intensity, as well as neutral (Kohler *et al.*, 2003), and participants are instructed to identify the expressed emotion among five possible choices.

Instead, the most reported test for assessing emotion discrimination ability is the Face Emotion Discrimination Test (FEDT), which was developed by Kerr and Neale (1993) in parallel with the FEIT. It uses 30 pairs of stimuli from the Izard (1971) set, and subjects are asked to decide whether the same or different emotions are presented. Like the FEIT, the FEDT is validated with established psychometric properties (Dougherty *et al.*, 1974; Feinberg *et al.*, 1986; Kerr and Neale, 1993; Zuroff and Colussy, 1986), although normative data are currently available only for USA and Korean (Bahk *et al.*, 2015) populations. Another test is the Emotion Differentiation Task (EMODIFF; Kohler *et al.*, 2000), in which participants are asked to differentiate the intensity of emotions shown in two side-by-side faces of the same person.

Few previous studies have combined these instruments and reported that both discrimination and recognition were impaired in patients and in high-risk individuals for psychosis (Addington *et al.*, 2008; Benito *et al.*, 2013; Comparelli *et al.*, 2013; Ulusoy *et al.*, 2020), suggesting a general impairment involving early stage of processing. On the other hand, studies with experimental paradigms assessing unconscious emotion processing, at behavioural and brain activity level, reported inconsistent data on early automatic stages, which resulted impaired or preserved in different samples of patients with psychosis (Brennan *et al.*, 2014; Gruber *et al.*, 2016; Williams *et al.*, 2009). The independency vs hierarchical dependency of the different stages including unconscious processing, early visual processing, recognition and labelling of emotions is a debated issue (Barrett *et al.*, 2007; Kring *et al.*, 2014). Hierarchical models propose a feed-forward propagation of signals from sensory to higher-level cortical areas, with the latter projecting feedback on early processing areas to support coherent representations throughout top-down modulation. Other models hypothesise the presence of independent pathways for conscious and unconscious processing, with signal diverging from early stages and involving distinct cortical and subcortical neural networks (Dehaene and Changeux, 2011; Tamietto and De Gelder, 2010). Within this framework, refining patients’ assessment has significant theoretical and translational implications. Individual perceptual sensitivity and unconscious processing can be evaluated with backward masking (Williams *et al.*, 2009) or continuous flash suppression (CFS) paradigms (Gruber *et al.*, 2016) to clarify whether emotional dysfunction is related to a deficit in early processing of salience and elucidate the role of anomalies in the recurrent interactions between visual and higher-level associative areas for access to consciousness (Dehaene and Changeux, 2011; Del Cul *et al.*, 2006).

In conclusion, emotion processing involves multiple stages, from unconscious processing to recognition and discrimination of emotions. Different tests, summarised in Table 1, have been developed to assess these stages. Although impairments have been observed in patients with non-affective and affective psychoses, the precise nature of these deficits remains a subject of debate. Therefore, refining patients’ assessment is crucial for a better characterisation of deficits in both affective and non-affective psychosis, which could be used to modulate new cognitive remediation and social skills training interventions aimed at enhancing emotion processing skills and improving effective interaction in social contexts.

**Table 1.** Characteristics of tasks used to assess the different stages of emotion processing in psychosis

Paradigm	Task	Authors	Features
Unconscious processing	CFS	Gruber *et al.*, 2016	Experimental task: binocular presentation of flashing stimuli.
	Backward masking (target <10 ms)	Williams *et al.*, 2009	Experimental task: the target stimuli are presented for less than 10 ms and followed by masking stimuli.
Perceptual sensitivity	Backward masking (varying target presentation)	Williams *et al.*, 2009	Experimental task: the target stimuli are presented varying their duration and followed by masking stimuli.
Discrimination	FEDT	Kerr and Neale, 1993	Validated test: 30 black and white pairs of faces.
	EMODIFF	Kohler *et al.*, 2000	Validated test: rate the emotional valence of 40 male and female faces displaying happy, sad or neutral expressions.
Recognition	EK-60F	Young *et al.*, 2002	Validated test: 60 black and white photographs of male and female faces expressing six basic emotions (surprise, happiness, fear, disgust, anger and sadness).
	FEIT	Kerr and Neale, 1993	Validated test: 19 black and white faces, 15 expressing anger, sadness, fear and shame; 4 expressing happiness and surprise.
	ER-40	Kohler *et al.*, 2003	Validated test: 40 faces expressing neutral, angry, sad, happy or fear emotions.

## Data Availability

All data used to write this paper are in the reference list.
